# A Fatal Case of a Foreign Body in the Air Passages

**Published:** 1862-04

**Authors:** John Bartlett

**Affiliations:** Pesotum, Ills.


ARTICLE XII.

### A FATAL CASE OF A FOREIGN BODY IN THE AIR PASSAGES.

By JOHN BARTLETT, M. D., Pesotum, Ills.

On the morning of the 20th of October last, a healthy female child of Mr. W. H., aged two and a half years, was seized with a violent fit of choking. After some minutes of extreme distress, during which a valvular sound was heard, as of a body moving to and fro in the windpipe, relief was obtained. Though there remained some wheezing, and, perhaps, some change in the color of the lips, the child sat up, and was disposed to engage in play. At night, the breathing becoming less quiet, and there being some restlessness, slight emesis was produced by a domestic remedy, and from that time the improvement was said to be constant, until eleven o'clock on the following night, when an examination of the case was made by me as complete as the extreme timidity and irritability of the patient would permit.

The mouth was closed, and the alae-nasi quiet; the color of the lips was good. The slight sigh at the close of expiration, noticed some hours previously, had ceased; the respiration, noiseless and easy, was 28, as the patient lay sleeping on the face: 30-32, when awake and semi-recumbent. The pulse was 139-140; the skin natural.

The respiratory movements of the two sides of the chest, as reviewed from behind, were alike in extent; but on the right side, the inspiration was attended by a significant *atmospheric forcing in* of the intercostal spaces, causing the ribs of this side to be better marked than their fellows opposite. On viewing the front of the chest, the conditions presented were not constant. Sometimes but little movement was noticed on the right side; the next moment no difference in the motion of the sides could be observed—and this without change of position.

Auscultation revealed, posteriorly on the right side, an imperfect murmur, rather tubular than vesicular; on the left side the respiration was exaggerated. At the root of the lungs no extraordinary sounds were detected.—Anteriorly the same general auscultatory phenomena obtained toward the median line, the puerile breathing of the left side obscured the blowing respiration of the right. In the right lateral region, a peculiar whistling, tubal sound was detected, in inspiration and expiration: immediately afterward, without change of position, during a full inspiratory effort, this sound gave place to the regular vesicular murmur.

At this time, the almost perfect quietude of the child had lulled the relatives into the false hope, that nothing had really passed into the windpipe. The evidence of the presence of a foreign body in the right bronchus was almost positive. It remained to determine, if possible, its nature. From a critical inquiry into the circumstances of the choking, it was unfortunately concluded that the body was a piece of a butter cake, which had been given to the child a few minutes before the accident. Being without the traditions of experience, as to the results, when substances liable to speedy disintegration, remain in the air passages, interference was deferred, that desired information might be obtained. In a few hours, I was hurriedly summoned. An effort at coughing had thrown the body from the bronchus, and the patient was dying of suffocation. Hearing upon entering the house that she still breathed, I opened the trachea, but the child was dead. Artificial respiration, with other restorative measures, persisted in for an hour, were without avail.

The offending substance proved to be a white bean, so swollen as nearly to fill the trachea, having an area in its small diameters of .083 inches. The left anterior jugular vein was large, and deviating to the right, lay immediately in the line of incision. No examination of the chest was allowed.

An important fact in physiology, and principle in practice, find illustration in this case, to wit:—

A foreign body may rest, for a time, in the air passages producing little or no constitutional disturbance.

The immediate opening of the windpipe is the only security for the patient in these cases; and all means for dislodging a body prior to the operation are unsafe.

March 24th, 1862.

---

A GIGANTIC SAURIAN.—The discovery of the fossil bones of a new and gigantic saurian, in a cutting recently made for a railway line near Poligny, has just been announced by M. Bertherand. The animal must have been between 90 and 118 feet in length, and must have existed towards the end of the Triassic period.

